# Behavioral Physiology of the CNTNAP2 Knockout Mouse

**Published:** 2025-05-28

**Authors:** Tanya Gandhi, Charles C. Lee

**Affiliations:** Department of Comparative Biomedical Sciences, LSU School of Veterinary Medicine, Baton Rouge, USA

**Keywords:** Autism, Mouse models, CNTNAP2, Repetitive behavior, Anxiety, Social behavior

## Abstract

Autism spectrum disorders (ASD) are composed of a range of conditions affecting the development of the nervous ststem; these groups of disorders are unified by characteristic impairments in social interaction, communication, and repetitive behaviors. Due to the somewhat hetereogeneous nature of these conditions, several environmental and genetic causes have been associated with ASD. Among the genetic causes, the CNTNAP2 gene is implicated in syndromic forms of ASD. As such, this gene has been studied in the context of animal model systems, primarily mice and rats; these studies have indicated that knockout of the CNTNAP2 gene results in features that are similar to that observed in human ASD. To further assess this animal model of ASD, in this study, we assessed the behavioral phenotypes of CNTNAP2 knockout mice using a range of behavioral assays targeting core and comorbid traits of ASD. We confirmed that CNTNAP2 mutant mice exhibited social interaction deficits, reduced ultrasonic vocalizations, repetitive behaviors, and hyperactivity, aligning with core and comorbid ASD features. Notably, we also identified novel findings: CNTNAP2 knockout mice displayed increased rearing behavior and lacked anxiety-like behaviors, diverging from the assumption that anxiety is a common feature of this model. These results demonstrate that CNTNAP2 knockout mice recapitulate key ASD-related behaviors while highlighting novel behavioral aspects; these data reinforce their utility as an animal model for studying ASD.

## Introduction

Autism spectrum disorders (ASD) are a group of behavioral disorders that arise due to altered patterns of neurodevelopment. The complex neurodevelopmental condition characterized by core behavioral patterns that result from atypical brain development41. The estimated prevalence of ASD has been attributed to several factors, including: ASD research, awareness among the public, health policies, and refined diagnostic criteria41; the prevalence currently varies between 60 to 100 per 10,000 children globally [1–3] and 1 in 54 cases in the US [3–8].

Early detection, diagnosis, and effective interventions are needed for those with ASD. Te diagnostic criteria for ASD relies on identifying a set of core behaviors: social interaction and communication deficits, and repetitive behaviors [9–12]. These behavioral traits are often accompanied by other symptoms such as: hyperactivity, anxiety, seizures, intellectual disabilities, and sleep disturbances [13,14]. The severity and prominence of both core and co-morbid features differ significantly among individuals with ASD [15]. Currently, the standard of care for ASD involves early behavioral interventions, which provides the most favorable outcomes [16,17]. Young children benefit from intensive behavioral therapy, which results in improved social skills and reduced repetitive behaviors. However, even the most prominent behavioral therapies are ineffective in many cases, expensive, time-intensive, and inaccessible in many areas. Thus, understanding gene-based behavioral phenotypes and causes of autism are crucial for developing targeted and effective medical therapeutics to alleviate symptoms and alter progression of the condition [7,18–21].

Autistic traits and related behaviors exhibit clinical heterogeneity and are linked to various genetic and environmental risk factors [22–24], with genetics factoring heavily in the etiology of autism [25,26]. Genetic mutations result in a higher likelihood of developing an autistic phenotype in monozygotic twins (92%) compared to dizygotic twins (10%) [20,27]. Numerous candidate genes have been identified as key contributors to the etiological heterogeneity of autism, influencing various neural development processes. Notably here, the CNTNAP2 (contactin-associated protein-like 2) gene is linked to a disorder known as cortical dysplasia focal epilepsy (CDFE); this condition is accompanied by autistic behaviors [28,29]. CNTNAP2 gene variations are correlated with increased risk of autism [28,30,31]. The CNTNAP2 gene encodes the neural transmembrane scaffolding protein, CASPR2 (contactin-associated protein-like 2), which is part of the neurexin superfamily of proteins [32,33]. This protein supports cruicial neural developmental processes, including the localization of voltage-gated potassium channels at the juxtaparanodal region of myelinated axons, the formation of dendritic spines, the mediation of neuron-glia interactions, and the organization of cortical layers [34–37].

Mice lacking the CNTNAP2 gene exhibit a range of behaviors that are typical of ASD, including social interaction and communication deficits, repetitive and stereotyped behaviors, and associated comorbid conditions such as hyperactivity, and seizures. Alongside the characteristic behavioral deficits observed in ASD, CNTNAP2 knockout mice display abnormal cortical neuronal migration and connectivity, asynchronous cortical neuronal firing, and an imbalance of cortical excitation and inhibition [38–42]. The altered cortical excitation and inhibition balance contributes to social behavior and information processing deficits [43]. Notable, CNTNAP2 mutant mice exhibit cortical overexpression of perineuronal nets that enwrap parvalbumin-positive interneurons. Digestion of these nets in the prefrontal cortex improves some social interaction deficits, indicating that regulating perineuronal nets and parvalbumin-expressing interneurons is crucial for maintaining excitation/inhibition balance and influencing social behaviors in neurological disorders like autism [40]. Further, these cortical neuroanatomical alterations can contribute to some of the autistic behaviors in this mouse model of the disorder [38,42,44,45]. While literature is available on behavior in the CNTNAP2 knockout mouse model, comparative studies conducting different behavioral tests on the CNTNAP2 mutant mouse strain are essential. Behavioral assays are influenced by both the genetic background of the experimental animals and specific experimental conditions [46,47].

Here, we demonstrate that the CNTNAP2^−/−^ knockout mouse model exhibits behaviors that are analogous to those in human ASD. The CNTNAP2^−/−^ mice display deficits in social interaction, communication, and stereotypic behaviors. In addition, we also performed behavioral tests evaluating ASD-associated co-morbid symptoms in the CNTNAP2^−/−^ mice. The CNTNAP2^−/−^ mice show hyperactive behavior as depicted by increases in locomotor activity. Hence, these data provide further validity of the autistic behavioral phenotype in the CNTNAP2^−/−^ mouse model of ASD that could be utilized to understand its role in brain development and function.

## Methods

### Animal Care and Housing

Wild-type (WT) C57BL/6J (strain: 000664) and mutant CNTNAP2 (strain: 017482) mice were obtained from the Jackson Laboratory (Bar Harbor, ME, USA). The offspring from these breeders were used for the study. All experimental protocols were approved by the Institutional Animal Care and Use Committee (IACUC) at Louisiana State University (Baton Rouge, LA, USA). The mice were group housed in a temperature- and humidity-controlled environment with a 12-hour light/dark cycle, lights on at 7:00 am, and had unlimited access to food and water.

### Behavioral assays

Mice underwent behavioral testing focused on core ASD domains, such as sociability and communication deficits, as well as repetitive behaviors. In addition, the mutant mice were also assessed for ASD-associated co-morbid behaviors. These procedures are similar to those that we have described previously [40].

### Three-chamber social interaction assay

The three-chamber social interaction test evaluated the time subject mice spend with stranger mice [48–50]. Two to three days before the test, mice were first acclimiated to the chamber for 30 min to 1 hour. Stranger mice were habituated in an inverted holding cup for 30 minutes daily over two days, ensuring they remained calm during testing. To prevent side bias, the placement of the stranger mice was alternated between the left and right portions of the chamber during habituation. The test consisted of three phases: Phase I involved 5 minutes of habituation in the center chamber with entry to the other chambers closed off. Phase II allowed 10 minutes for exploration of all empty chambers. In Phase III, during the sociability test, the subject mice was allowed 10 minutes to expore the chamber: one side (labeled ‘E’) featuring an empty cup and the other side (labeled ‘S1) held a gender-matched stranger mouse (C57BL/6J). As a control for side preferance, the stranger mouse position was switched between the two sides. The time spent interacting with the stranger mouse was analyzed using ANY-maze software (Wood Dale, IL, US), and the percentage of time spent with the stranger mouse (S1) was calculated as [(S1 / (S1 + E)) × 100].

### Recording USV in pups

Ultrasonic vocalizations emitted by rodent pups were used for assessing vocal communication [51]. Pups emit calls more frequently upon separation from the home cage and dam. These repeated vocalizations elicit maternal retrieval behavior in the dam. USVs were elicited in rodent pups, leading to robust data collection in a short period of time. A sound attenuating chamber was used for the recording of vocalizations with a microphone attached to the unit. A mouse pup was held in a glass beaker located inside a chamber that attenuated sound. An ultrasonic microphone (#AT125, Binary Acoustics, PA, US) was placed above the beaker to record the calls. The vocalizations were recorded using the SPECT’R software (Binary Acoustics, PA, US) for 10 min. SCAN’R software (Binary Acoustics, PA, US) was used to analyze the recordings. In the SCAN’R software, the cut-off frequency was set at 30kHz, filtering out the background noise, and the minimum duration of calls was set at 5ms. The calls were recorded and analyzed at different postnatal age groups such as postnatal day (PD) 4, 6, 8, 10, and 12. At the end of the recording, the pup was removed, weighed, tail marked, and returned to the home cage. The measures evaluated include call rate (number of calls per minute), and body weight.

### Self-grooming test

Rodent self-grooming is an inherent behavior. Over- and under-grooming is a manifestation of genetic mutations, stress, and altered gene-environment interactions, as previously described [52]. This behavioral test was always conducted in the afternoon between 14:00 and 18:00 h. Habituation of the animals was performed in the testing room, 3 days before the test (2–3 hours) and then again on the test day (30 min before starting). During the test, the first 10 min were recorded as baseline habituation assessment; the next 10 min were used to assess self-grooming behavior. Videos of grooming behavior were captured using a camera that was located above the cage (1080 HD, Logitech, CA, USA). 70% ethanol was used to clean the chamber between tests to eliminate any residual odors. Manual analysis of videos was performed in a randomized fashion using MATLAB software (MathWorks, US). The spontaneous self-grooming behavior was analyzed during the different developmental (postnatal) age groups (PD18, PD32, and PD60). Motor stereotypies observed include repetitive self-grooming and vertical rearing i.e., time spent climbing and/or jumping on the side walls of the test apparatus.

### Y-maze spontaneous alteration test

Mice naturally explore new environments i.e., exploratory behavior. In the Y-maze test, mice typically tend to investigate a new arm of the maze rather than returning to a previously visited arm, as we previously described [53]. We employed a custom-made Y-shaped maze with three identical arms connected to each other at an angle of 120°. The mice were placed in the center of the Y-maze with free access to all three arms. The mice freely explored all three arms of the Y-maze for 5 min. The spontaneous alterations were measured as the percentage of alterations by recording the number of arm entries and sequence of entries. An arm entry was measured when all four limbs were within the arm (85% of the body in the arm). A spontaneous alteration was scored when a mouse entered a different arm of the maze (no repetition) in a sequence of arm visits i.e. A to B to C. Spontaneous alteration % was calculated as follows:

$$\text{Spontaneous}\;\text{alteration}\%=\frac{\#\text{spontaneous}\;\text{alterations}\;\text{X}\;100}{\text{total}\;\text{number}\;\text{of}\;\text{arm}\;\text{entries}-2}$$

### Open-field test

The open-field maze assess the inherent mouse behavior to avoid open spaces [54]. We used a sqaure maze with dimensions of 40 × 40 × 30 (l×w×h in cm; Maze Engineers, Boston, MA). Before testing, acclimation of mice in the testing room was performed for 30–60 min. As above, 70% ethanol was used to clean between tests. Video recording was performed as noted above. Furing the test, the mouse was able to explore the area for 5 min. The video data files were analyzed in ANY-maze (Stoelting). Using the software, the test area was divided into 5 × 5 square grids for analyzing the travel distance traveled, entries and time spent in center and periphery regions, time spent immobile, and fecal boli count.

### Light-dark Exploration Test

The light-dark exploration test is used to assess anxiety-like behaviors in rodent models, as previously described [55]; rodents prefer the dark compartment and avoid the illuminated light compartment. The light-dark apparatus was made up of a rectangular box divided equally into light and dark compartments, which were made of black and white acrylic sheets. The mice were acclimated to the testing room 30–60 min before the test. The mouse was started in the light side compartment and explored both chambers for 5 min. Video was recorded as noted above. ANY-maze ANY-maze (Stoelting) was used to measure the total time and entries in the dark and light sides of the chamber.

### Forced Swim test

The forced swim test is commonly used to assess depressive-like and stress-coping behaviors, as described previously [56]. We used a glass beaker (5L) filled with water to a depth of 20 cm. A mouse was placed into the beaker and allowed to swim freely for 5 min. Video was recorded using a camera (1080 HD, Logitech, CA, USA) located on the side of the beaker. Two mice were tested simultaneously using a black cardboard sheet to block each other’s view. The immobility time was then determined from the recorded videos.

### Statistical Analysis

Data were analyzed using Student’s t-test to determine significant differences among groups, with a p-value of <0.05 considered statistically significant. Statistical analysis was performed using GraphPad Prism 5 (GraphPad Software, La Jolla, CA).

## Results

As ASD diagnostic criteria primarily depend on the characterization of behavioral symptoms, we performed behavioral tests relevant to core ASD behavioral domains and associated co-morbid symptoms [38].

### Social interaction behavior deficits

**Figure 1:**
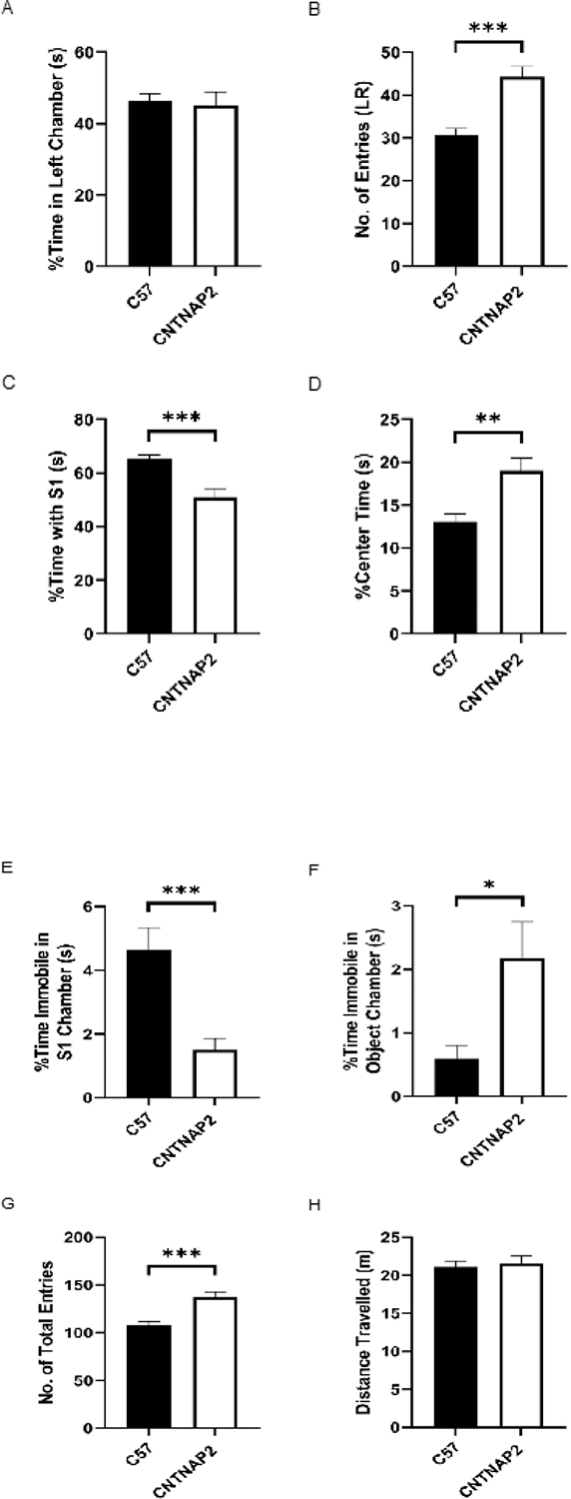
Three-chamber sociability performance in mice. (A, B) no chamber preference between the groups and increased entries in both the left and right chamber of the three-chamber test apparatus in the CNTNAP2−/− group than the C57BL/6J group during the habituation phase of the social interaction test (C, D) CNTNAP2−/− mice spent less time with the stranger mice and spent more time in the center chamber as compared to the C57BL/6J mice (E, F) Decreased time spent immobile in the chamber housing stranger mouse and increased time spent immobile in the chamber housing object in the CNTNAP2−/− group compared to the C57BL/6J group (G, H) Increased number of total entries in all the three chambers of the test apparatus in the CNTNAP2−/− group as compared to the C57BL/6J group and no change in the total distance traveled between the groups. C57BL/6J (n=20) and CNTNAP2−/− (n=22) mice. Data expressed as mean ± SEM (p<0.05).

Social behavior in Cntnap2 mutant mice was conducted with the three-chamber social interaction test in adult CNTNAP2^−/−^ and C57BL/6J mice [48]. In this test, the habituation data (phase II test) confirmed the lack of chamber preference in the control and test mice (i.e., the subject spends similar time in the left and right chambers this confirmed that the room cues were well balanced before moving on with sociability (phase III) tests [48]. The number of entries in the two sides of the chamber was significantly increased in the CNTNAP2 KO mice indicating increased locomotion. The CNTNAP2^−/−^ mice showed a sociability deficit, spending less time with the stranger (S1) mouse than the control (E) mice. Additionally, the time spent in the center chamber was greater in the mutant group than in the WT group. Further, the mutant group spent more time immobile in the chamber with an empty object compared to the chamber housing stranger mice, indicating abnormal social behavior in the mutant strain. The CNTNAP2^−/−^ mice also exhibited hyperactive behavior as observed by more total entries in the 3-chamber apparatus compared to the WT mice. Total distance traveled did not differ between the groups during the test (Figures 1A–1H).

### Impairments in communication

**Figure 2:**
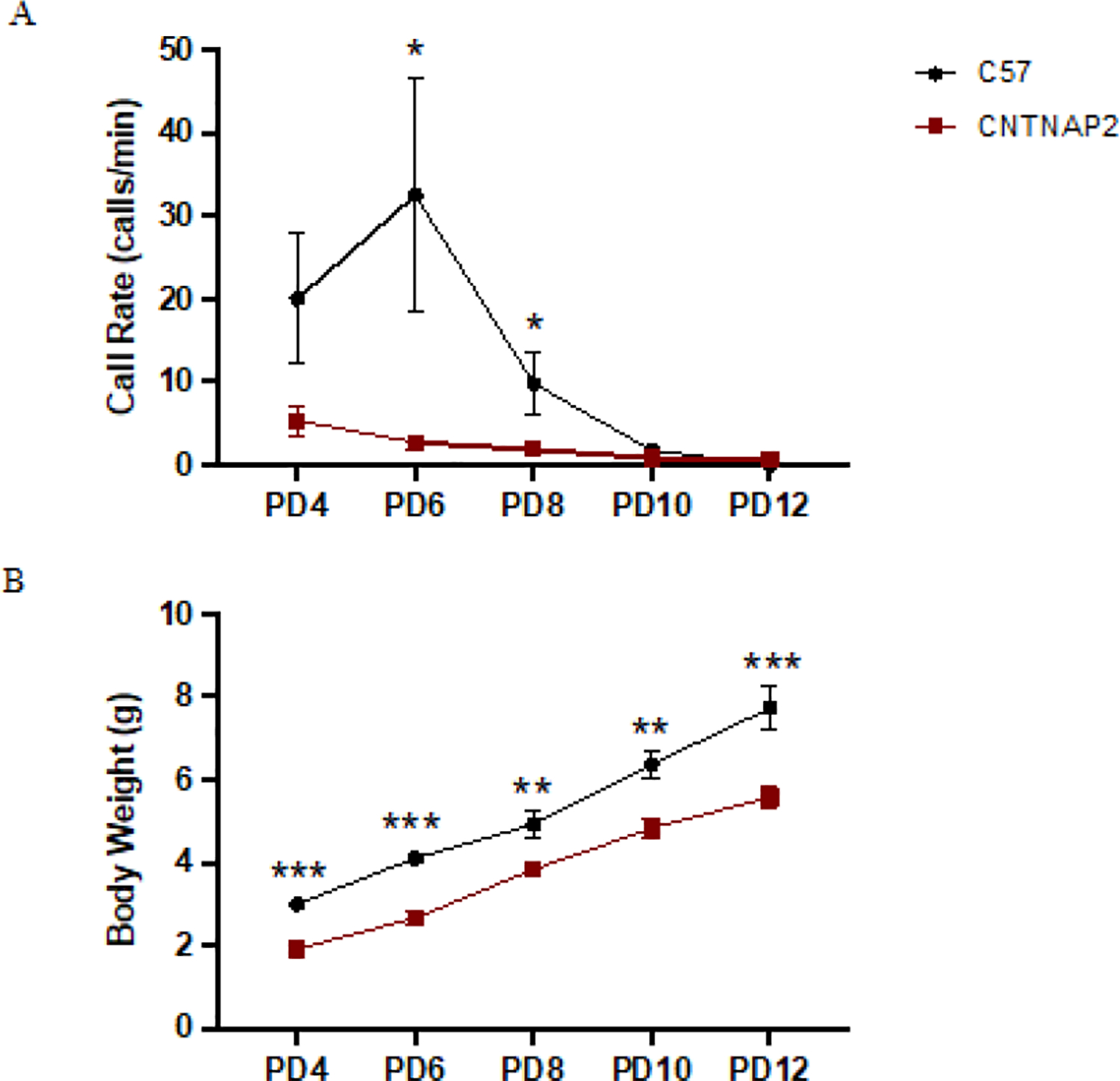
Ultrasonic vocalizations emitted by the mouse pups. (A) Calls emitted per minute by the CNTNAP2−/−, and C57BL/6J pups across different postnatal ages (B) Body weight measure of CNTNAP2−/− and C57BL/6J pups across various postnatal ages. C57BL/6J PD4 - PD10 (n=10–12); PD12 (n=8), and CNTNAP2−/− PD4 - PD10 (n=11–12); PD12 (n=5) mice. Data expressed as mean ± SEM (p<0.05).

ASD mouse models typically exhibit altered ultrasonic vocalizations (USVs) [57,58]. The communication deficit in mutant mice was assessed by recording the USVs elicited by the pups following maternal separation. These vocalizations are distress calls produced by the pups to elicit maternal retrieval behavior that is a necessary component in normal infant-mother interactions; this is critical for infant growth and development and potentially relevant to ASD [38,46,59–61]. We examined USVs in mutant and WT mice across different postnatal (PD) developmental ages (PD4, PD6, PD8, PD10, and PD12). The CNTNAP2^−/−^ pups after separation from the dam and the littermates elicited calls less frequently as compared to the C57 control pups at different postnatal ages, confirming communication deficit in the CNTNAP2^−/−^ mice early in the development (Figures 2A, 2B). This reduction in mutant pup calls paralleled the decrease in body weight of the mutant group as compared to the control group at different developmental ages. Previous observations in CNTNAP2 mutant mice have shown reduced vocalizations and auditory processing impairments, indicative of abnormal communication in the mutant mice [38,62].

### Repetitive and stereotyped behaviors

**Figure 3:**
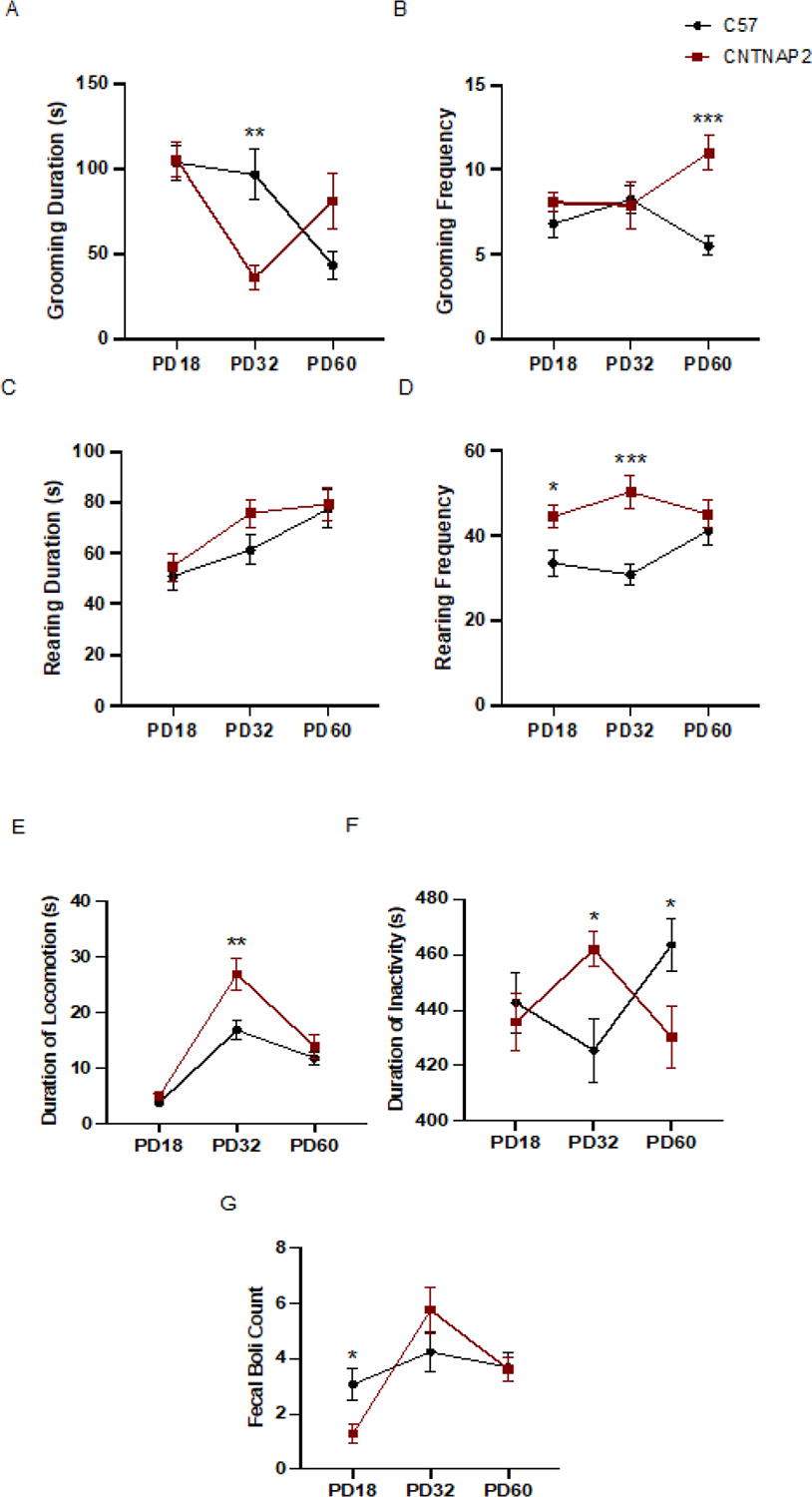
Repetitive self-grooming measures at PD18, PD32, and PD60 (A, B) self-grooming duration and frequency, (C, D) vertical rearing duration and frequency, (E, F) duration of locomotion and inactivity, and (G) fecal boli count in CNTNAP2−/−, and C57BL/6J mice. C57BL/6J (n=15–22), and CNTNAP2−/− (n=12–18) mice. Data expressed as mean ± SEM (p<0.05).

Individuals with ASD exhibit motor stereotypies and impairments in behavioral flexibility including repetitive behaviors [63–65]. The CNTNAP2^−/−^ mice showed a decrease in grooming duration at PD32 compared to C57 mice. However, the CNTNAP2^−/−^ mice showed increased grooming frequency (grooming bouts) at PD60 with respect to control mice. There was no difference in the rearing duration between the groups at any of the postnatal ages. The CNTNAP2^−/−^ mice demonstrate enhanced rearing frequency (number of vertical jumps) at PD18 and PD32 than the control group. Further, the CNTNAP2^−/−^ mice showed an increase in the duration of locomotion at PD32 and a decrease in the duration of inactivity compared to control mice at PD60, indicating hyperactivity in the mutant group. However, the CNTNAP2^−/−^ mice showed an increase in duration of inactivity than the mice at PD32. This increase in both locomotion and inactivity observed at postnatal day 32 can be explained by behavioral rigidity in the mutant strain affecting overall activity patterns, leading to increased hyperactivity followed by periods of extended inactivity [38,66,67]. These observations indicate stereotypic behaviors manifested by the CNTNAP2^−/−^ mice at various developmental ages. Lastly, the CNTNAP2 mutant group had less fecal boli count as opposed to the group at PD18, but fecal boli count did not differ between groups at other postnatal ages (Figures 3A–3G).

**Figure 4:**
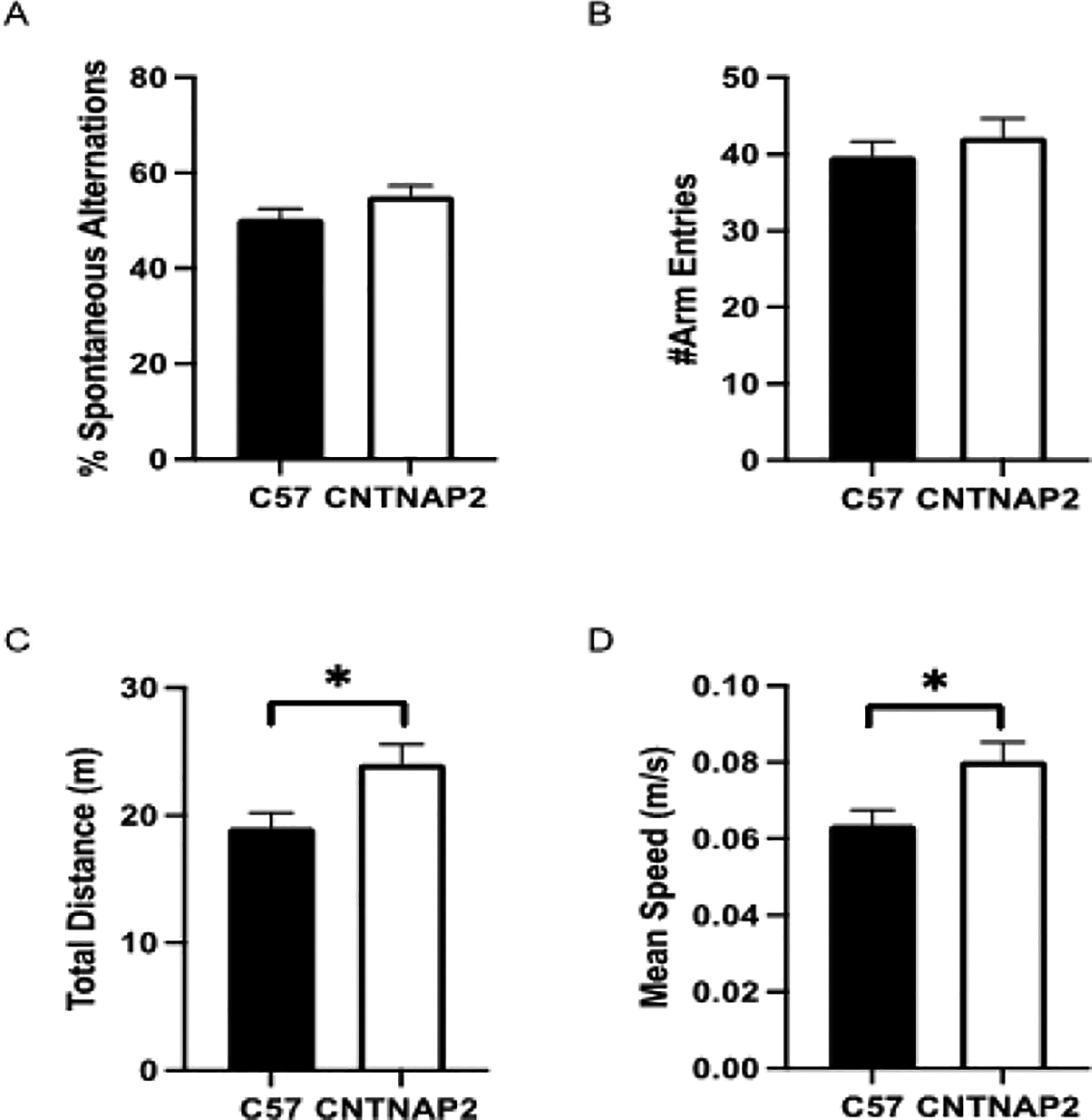
Y-maze spontaneous alternations test data measures (A) the percentage of spontaneous alternations, (B) the number of total arm entries, (C) the total distance traveled, and (D) the mean speed in CNTNAP2−/− and C57BL/6J mice. C57BL/6J (n=18), and CNTNAP2−/− (n=18) mice. Data expressed as mean ± SEM (p<0.05).

Parameters such as exploratory behavior and spatial working memory can be measured in the Y-maze test. Mice are exploratory in nature and tend to explore all aspects of their new environment. This innate exploratory behavior was assessed using the Y-maze, in which the mice explored different arms of the maze [53,68]. Further, a Y-maze spontaneous alternation test was performed to assess perseverative tendency and behavioral inflexibility. CNTNAP2^−/−^ mice exhibited an increase in total distance traveled and the mean speed in the Y-maze spontaneous alternations test. No change was observed in the spontaneous alternations as well as the number of total entries into different arms of the Y-maze between the groups (Figures 4A–4D).

### ASD-linked comorbidities: hyperactivity, anxiety, and depression-like behavior

**Figure 5:**
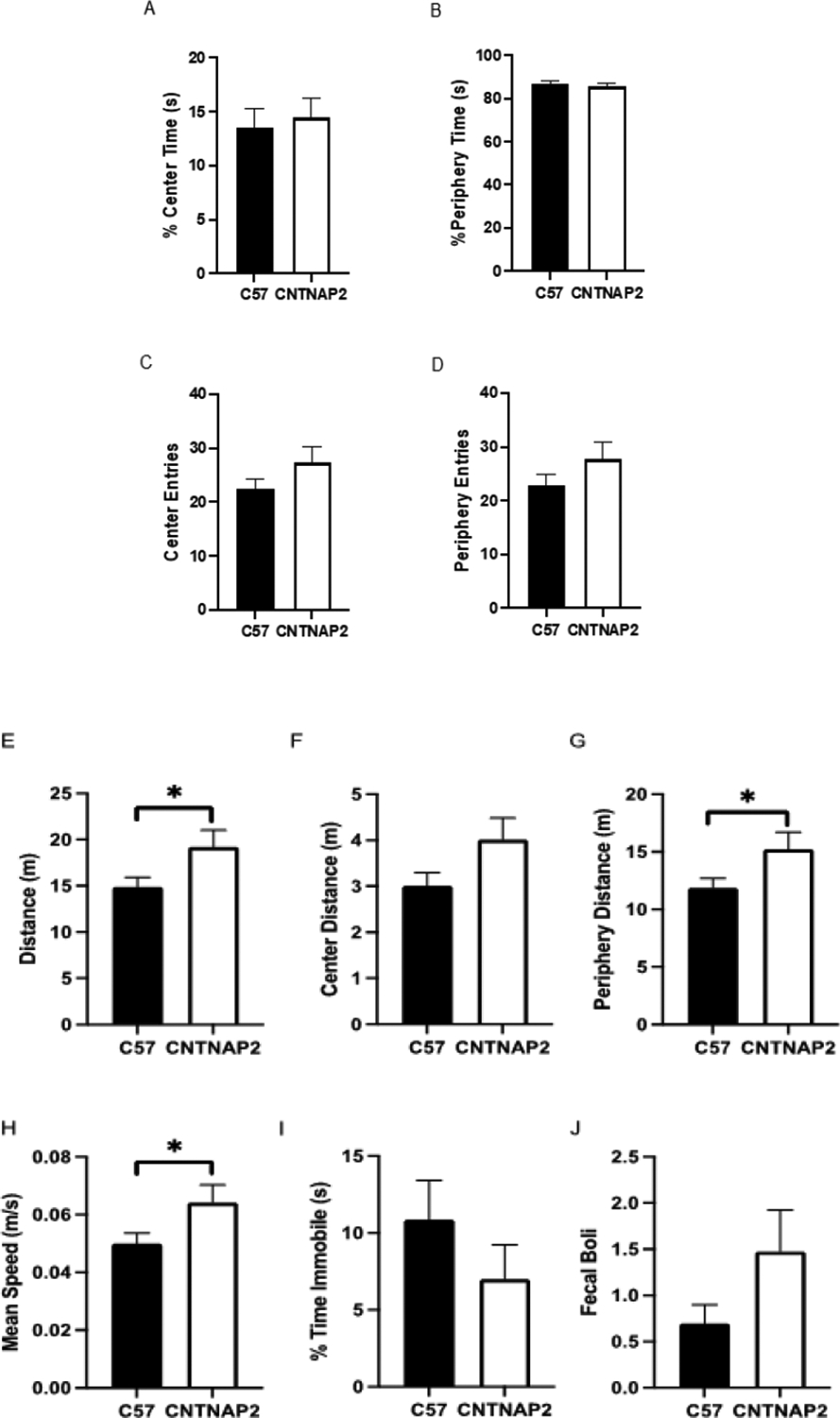
Open-field test data measures (A) the percentage of time spent in the center, (B) the percentage of time in the periphery, (C) the number of entries in the center, (D) the number of entries in the periphery, (E) the total distance traveled, (F) the total distance traveled in the center, (G) the total distance traveled in the periphery, (H) the mean speed, (I) the percentage of time spent immobile, and (J) the fecal boli count in CNTNAP2−/− and C57BL/6J mice. C57BL/6J (n=23), and CNTNAP2−/− (n=19) mice. Data expressed as mean ± SEM (p<0.05).

Mice were then assayed to detect potential confounding factors. Associated co-morbid symptoms that occur in some autistic individuals include hyperactivity, anxiety, hyper-reactivity and hypo-reactivity to sensory stimulation, seizures, and gastrointestinal problems [38]. We conducted open-field and light-dark exploration tests to measure anxiety-related behavior and locomotor activity. In the open-field test, there was no difference among groups in the total time spent and the number of total entries in the periphery and center areas of the open-field apparatus, suggesting an absence of open-space anxiety-related behavior in the mutant group (Figures 5A–5J). However, the CNTNAP2^−/−^ mice exhibited increases in total distance traveled, total peripheral distance traveled, and mean speed, suggesting greater locomotor activity than their WT littermates in the open field test.

**Figure 6:**
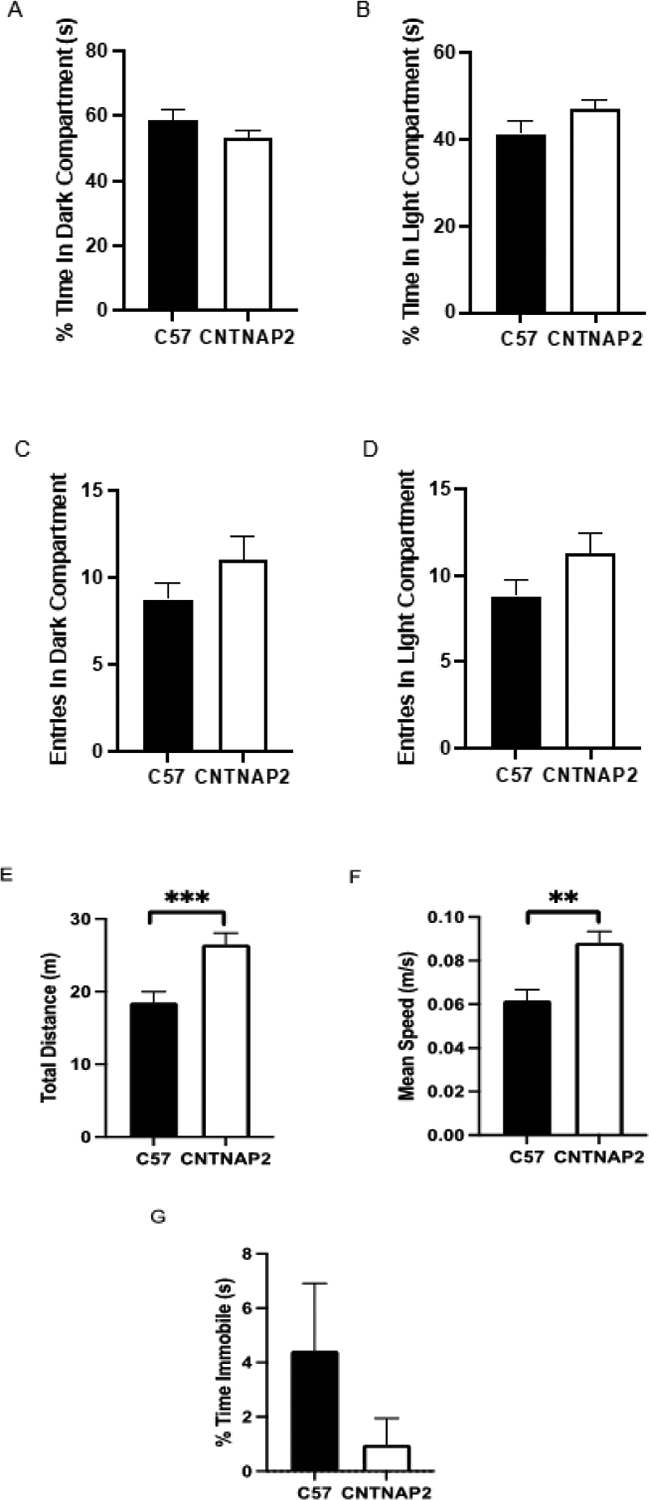
The dark-light box test measures (A) the time spent in the dark compartment, (B) the time spent in the light compartment, (C) the total number of entries in the dark compartment, (D) the total number of entries in the light compartment, (E) the total distance traveled, (F) the mean speed, and (G) time spent immobile in the test apparatus in CNTNAP2−/− and C57BL/6J mice. C57BL/6J (n=17), and CNTNAP2−/− (n=16) mice. Data expressed as mean ± SEM (p<0.05).

Furthermore, to assess anxiety-related responses, we performed the light-dark transition test that measures the aversion of mice to brightly lit spaces, indicating bright-space anxiety [69]. There was no difference between the CNTNAP2^−/−^ and C57 groups in the amount of time spent in the light and dark chamber of the light-dark test apparatus, which confirms that the CNTNAP2 mutant mouse model of autism does not show anxiety-related behavior. Additional measures of the light-dark test indicate an increase in total distance traveled, and mean speed with no change in time spent immobile among groups (Figures 6A–6G).

**Figure 7:**
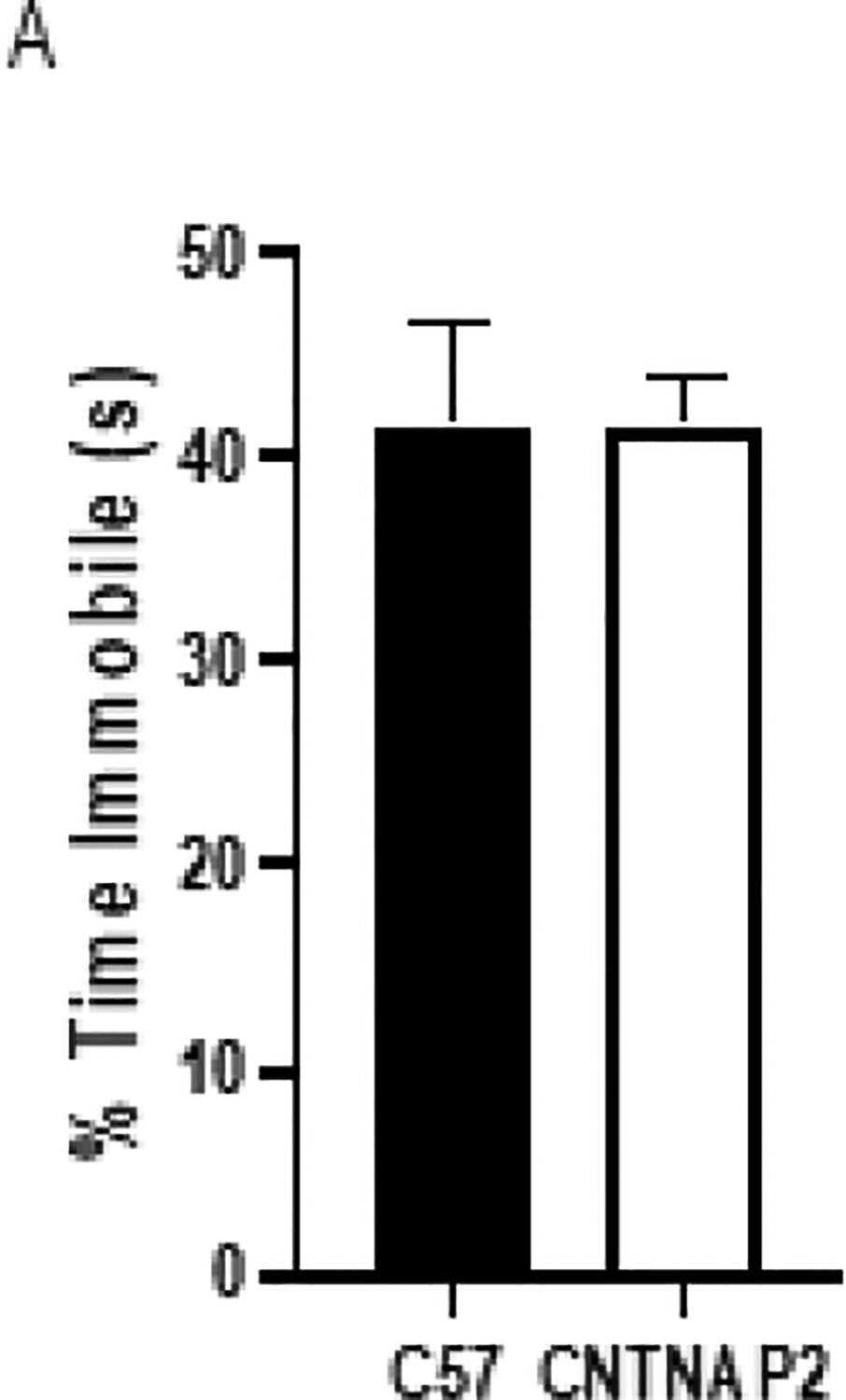
Forced-swim test measure (A) the time spent immobile in CNTNAP2−/− and C57BL/6J groups. C57BL/6J (n=12), and CNTNAP2−/− (n=12) mice. Data expressed as mean ± SEM (p<0.05) (p<0.1).

The depressive-like behavior in mice was measured using the forced swim behavioral test. Depressive behavior correlates with altered response to stress and difficulty in adapting to a change. No difference was observed between groups in the amount of time spent immobile (Figure 7A), indicating no stress-related depressive behaviors in the CNTNAP2 mutant group.

## Discussion

The etiology of ASD is extremely heterogeneous [22,24]. Behavioral testing in animal models, particularly in mice harboring gene mutations relevant to autism, is crucial for advancing knowledge and understanding mechanisms contributing to such behavioral symptoms. Such models facilitate the investigation of how specific genetic alterations influence behavior and neurodevelopment, shedding light on the mechanisms underlying disorders like autism^46,49^. Evaluating behaviors such as social interaction, communication, and repetitive actions, can help discern the phenotypic effects of genetic mutations [70,71]. Behavioral assessment studies in mice not only contribute towards elucidating the pathophysiology and underlying neural mechanisms of neurodevelopmental disorders but also support the assessment of potential therapeutic interventions [19,61]. Additionally, behavioral testing in genetically modified mice allows for the examination of how targeted treatments might alleviate or reverse abnormal behaviors, thereby contributing to the development of effective management and treatment strategies [20,61].

Our study builds upon existing behavioral phenotyping of the CNTNAP2 mutant mice, providing additional insights and expanding understanding of their behavioral repertoire. Here, we show that CNTNAP2 knockout mice exhibit many of the behavioral features observed in patients with idiopathic autism and with recessive CNTNAP2 mutations that cause a syndromic form of autism [34]. Cntnap2 KO mice have normal anxiety-related responses but show abnormal vocal communication, repetitive behaviors, and abnormal social interactions [38]. In addition, they also show hyperactivity which is a co-existing feature in both CDFE patients and in many patients with ASD.

Social interaction deficits are a core behavior in ASD. Mice, being a highly social species, naturally engage in behaviors such as socially investigating unfamiliar conspecifics, communal nesting, group huddling for sleep, parental care, and juvenile play [72–74]. The sociability test measures the inclination to approach and socially investigate another mouse. Previous studies have shown significant correlations between the time spent sniffing and the time spent in the chamber with the stranger mouse, indicating that the normal subject mice prefer to interact with the stranger mouse [75–77]. Hence, in this study reduced time spent in the chamber with the stranger mouse confirmed social interaction deficits in the CNTNAP2 mutant mice model.

Impaired social communication is also a core feature of ASD [78,79]. Although mice do not vocalize in a language-like manner, they do exhibit robust social communication mechanisms, including the emission of ultrasonic vocalizations. A well-known example of social communicative behavior in rodents is the ultrasonic vocalization produced by pups when they are away from the nest. These ultrasonic “distress” calls prompt the parents to locate and return the pup to the nest. Reduced levels of these infant vocalizations may be indicative of the communication deficits seen in autism [61,80–82]. In this study, reduced vocalizations were observed in the CNTNAP2 mutant mice pups, suggesting a communication deficit at an early phase of life in the mutant group. These findings are consistent with previous research linking CNTNAP2 mutations to impaired social communication, further validating the role of this gene in early neurodevelopmental deficits.

Autistic individuals often maintain rigid, ritualistic routines, and display repetitive behaviors [82]. Compulsive behaviors are also observed in ASD mice models [83]. Notably, repetitive behaviors manifest as persistent grooming, jumping, sniffing, circling, digging, and continuous cage-running [84]. Self-grooming is an innate behavior in animals, essential for hygiene, and other physiologically important processes, and is remarkably consistent across species [85–87]. While humans also engage in self-grooming, it can become pathological under stress or in certain neuropsychiatric disorders [88–91]. Rodent self-grooming, therefore, serves as a valuable indicator for complex, repetitive, and self-directed behaviors, offering insights into the neural basis of complex behavioral regulation in both normal and abnormal conditions, which may be relevant to understanding similar behaviors in humans [87]. Self-grooming in rodents mirrors pathological repetitive behaviors like behavioral perseveration; thus studying rodent strains that display these traits is essential for identifying neural circuits and genes linked to ASD [92,93]. In the grooming behavioral analysis, we found that the CNTNAP2 mutant mice exhibited repetitive and stereotypic behaviors, such as increased grooming frequency. This finding aligns with previous research that has demonstrated increased grooming and stereotypic behaviors in CNTNAP2 mutant animals [83]. Notably, our study also observed increased rearing frequency in CNTNAP2 mutant mice. However, in another study, no significant difference was found in rearing in CNTNAP2^−/−^ mice [94], but CNTNAP2^−/−^ rats displayed increased rearing [95]. The grooming and rearing behaviors were assessed in an empty standard mouse cage, which may have influenced the observed behavioral differences, which differs from other studies conducted in open-field arenas. Hence, our results add new insights by demonstrating increased rearing in CNTNAP2^−/−^ mice.

In terms of locomotion, our findings highlight heightened locomotor activity across various tests. In the Y-maze spontaneous alternation test, the CNTNAP2^−/−^ mice exhibit an increase in total distance traveled and mean speed with no considerable difference in spontaneous alternations or number of total arm entries. This combination of increased locomotion and unchanged alternation behavior indicates that the CNTNAP2^−/−^ mice exhibit hyperactivity and stereotypy without any behavioral inflexibility in this specific test. This heightened locomotion was consistent across other tests, including the open-field and dark-light box tests, where CNTNAP2^−/−^ mice exhibited increased exploratory movement. These findings are consistent with hyperactivity rather than anxiety, as CNTNAP2^−/−^ mice did not exhibit behaviors typically associated with anxiety, such as freezing or avoidance in the light-dark box or forced swim tests. This aligns with previous studies reporting increased locomotor activity in mouse models of autism [96–99]. However, in another study, no significant difference was found in the total distance traveled in CNTNAP2^−/−^ mice^94^, but CNTNAP2^−/−^ rats displayed hyper-locomotion [95]. These findings may reflect species-specific differences or the unique testing conditions employed in this study. Ultimately, the heightened locomotor activity across various tests supports the notion that hyperactivity, rather than anxiety, is a characteristic of the CNTNAP2^−/−^ autism model associated with autistic behavioral traits.

The fecal boli count in mice is commonly used as an indicator of anxiety or stress-related behaviors in rodent models. Increased defecation during behavioral tests is often interpreted as a physiological stress response, with higher fecal boli counts generally correlating with elevated anxiety and heightened emotional arousal in mice [100]. In this study, we observed no significant difference in fecal boli count between the CNTNAP2^−/−^ mice and controls, except at PD18 during the grooming test, where controls released more fecal boli, which could be attributed to the younger age of the mice at the time of testing. This suggests that the knockout mice do not exhibit heightened anxiety or stress during the grooming and open-field tests. This finding is novel, as it demonstrates that despite exhibiting core autism-related behaviors—such as altered social interactions, repetitive behaviors, communication deficits, and hyperactivity—the CNTNAP2^−/−^ mice do not display elevated anxiety based on this measure. This dissociation between autism-related traits and anxiety in CNTNAP2^−/−^ mice challenges the assumption that anxiety always co-occurs with these traits and underscores the complexity of behavioral phenotyping in this model. The lack of difference in fecal boli count suggests that anxiety may not be a primary or distinguishing factor in this model, highlighting a unique aspect of the CNTNAP2^−/−^ behavioral phenotype.

As autism is diagnosed based on behavioral rather than physiological criteria, the validity of mouse models for this disorder relies on their ability to accurately reflect core behavioral symptoms, such as social interaction impairments, communication deficits, and stereotypic behaviors. Comprehensive behavioral phenotyping is a crucial strategy to identify promising mouse models for further genetic characterization of ASD. The CNTNAP2 knockout mouse model, through the behavioral alterations observed in this study, provides valuable insight into these core symptoms [101–103]. These mutant mice are thus a viable animal model for understanding the underlying neural mechanisms, and pharmacological and biological targets to develop therapeutic interventions aimed at the primary symptoms of ASD [38,46,70,104–107]. Such mouse models have shown encouraging results [41,108], providing evidence that different neural pathways contribute to the ASD-related core behavioral features. This study supports the use of the CNTNAP2 mouse model for testing new therapeutic strategies and strengthens the existing behavioral data on CNTNAP2 mutant mice, laying a foundation for future investigations into the neural mechanisms underlying ASDs and the development of potential therapeutic approaches.

## Conclusion

Rodent models can advance our understanding of disease mechanisms and evaluating the potential efficacy of therapeutic interventions. In this study, the behavior of CNTNAP2 knockout and C57BL/6J wildtype mice were compared, confirming several behaviors previously reported in the literature. CNTNAP2 knockout mice exhibited social interaction deficits, impaired communication, increased grooming, and hyperactivity—behaviors consistent with prior findings. Notably, our study also identified novel results, including increased rearing behavior and a lack of anxiety in the CNTNAP2 knockout mice. These findings suggest that while CNTNAP2 knockout mice replicate key ASD-related behaviors such as social interaction deficits and repetitive behaviors, anxiety may not be a distinguishing feature in this model. Continued behavioral evaluation of such models is essential for gaining comprehensive insights into the disorder and uncovering neural pathways and mechanisms that could lead to targeted therapies for neurological and neuropsychiatric disorders.
